# Evaluating psychiatric case-control studies using the STROBE (STrengthening the Reporting of OBservational Studies in Epidemiology) statement

**DOI:** 10.1590/1516-3180.2014.1323653

**Published:** 2014-04-28

**Authors:** Pedro Domingues Goi, Julia Domingues Goi, Kariny Larissa Cordini, Keila Mendes Ceresér, Neusa Sica da Rocha

**Affiliations:** I MD, MSc. Doctoral Student, Postgraduate Medical Program on Psychiatry, Universidade Federal do Rio Grande do Sul (UFRGS), Porto Alegre, Brazil; II Pharm, MSc, PhD. Professor, Postgraduate Medical Program on Psychiatry, Universidade Federal do Rio Grande do Sul (UFRGS), Porto Alegre; Member, INCT for Translational Medicine, Brazil; III MD, MSc, PhD. Professor, Postgraduate Medical Program on Psychiatry, Universidade Federal do Rio Grande do Sul (UFRGS), Porto Alegre, Brazil

**Keywords:** Epidemiology, Psychiatry, Research design, Case-control studies, Biomedical research, Epidemiologia, Psiquiatria, Projetos de pesquisa, Estudos de casos e controles, Pesquisa biomédica

## Abstract

**CONTEXT AND OBJECTIVE:**

Case-control studies are important in developing clinical and public health knowledge. The STROBE statement (STrengthening the Reporting of OBservational Studies in Epidemiology) was developed to establish a checklist of items that should be included in articles reporting observational studies. Our aim was to analyze whether the psychiatric case-control articles published in Brazilian journals with CAPES Qualis rating B1/B2 in 2009 conformed with the STROBE statement.

**DESIGN AND SETTING:**

Descriptive study on psychiatric papers published in Brazilian journals, within the Postgraduate Medical Program on Psychiatry, at Universidade Federal do Rio Grande do Sul.

**METHODS:**

All psychiatric case-control studies from Brazilian Qualis B1/B2 journals of psychiatry, neurology and public health in 2009 were analyzed. The four most specific items of the STROBE statement were used to evaluate whether these studies fitted within the case-control parameters: 1) selection of cases and controls; 2) controlling for bias; 3) statistical analysis; and 4) presentation of results.

**RESULTS:**

Sixteen case-control studies were identified, of which eleven (68.75%) were in psychiatry-focused journals. From analysis using the STROBE statement, all of the articles conformed with item 1; two (12.5%) completely conformed with item 2; none completely conformed with item 3; and only three (18.8%) conformed with item 4.

**CONCLUSION:**

The case-control studies analyzed here did not completely conform with the four STROBE statement items for case-control design. In view of the inadequate methodology of the published studies, these findings justify focusing on research and methodology and expanding the investigations on adherence of studies to their designs.

## Abstract (pt)

**CONTEXTO E OBJETIVO:**

Estudos de caso-controle são importantes no desenvolvimento do conhecimento clínico e de saúde pública. O STROBE statement (STrengthening the Reporting of OBservational Studies in Epidemiology) foi criado para estabelecer uma lista de itens que devem estar presentes na descrição de estudos observacionais. Nosso objetivo é analisar a adequação de artigos caso-controle psiquiátricos publicados em periódicos brasileiros Qualis B1/B2 CAPES em 2009 utilizando o STROBE statement.

**TIPO DE ESTUDO E LOCAL:**

Estudo descritivo de artigos em psiquiatria publicados por periódicos brasileiros, realizado no Programa de Pós-Graduação em Medicina: Psiquiatria, na Universidade Federal do Rio Grande do Sul.

**MÉTODOS:**

Todos os estudos de caso-controle psiquiátricos em revistas brasileiras Qualis B1/B2 de psiquiatria, neurologia e saúde pública em 2009 foram analisados. Os quatro itens mais específicos do STROBE statement foram utilizados para avaliar se os estudos se ajustavam aos parâmetros de caso-controle: 1) seleção de casos e controles, 2) controle de vieses, 3) análise estatística e 4) apresentação dos resultados.

**RESULTADOS:**

Dezesseis estudos de caso-controle foram identificados, 68,75% (11) deles de periódicos de psiquiatria. Após a análise com base no STROBE statement, todos os artigos adequavam-se ao item 1; 12,5% (2) adequavam-se completamente ao item 2; nenhum ajustava-se completamente ao item 3; e somente 18,8% (3) estavam adequados em relação ao item 4.

**CONCLUSÃO:**

Os estudos de caso-controle avaliados aqui não se adequaram completamente aos quatro itens do STROBE statement para o desenho de caso-controle. Tendo em vista a inadequada metodologia dos estudos publicados, os achados justificam direcionar-se o foco para a pesquisa e metodologia, aumentando a investigação da adesão dos estudos aos seus desenhos.

## INTRODUCTION

Rational healthcare practice requires knowledge of the etiology, pathogenesis, diagnostics, prognosis and treatment of illnesses. A substantial portion of clinical and public health knowledge comes from observational research. Nine out of ten articles published in clinical journals describe observational investigations.^1^

Case-control studies belong to the observational studies group. They emerged within epidemiology as part of the search to identify the risk factors in diseases. A case-control design is modest and less expensive than other models, and can produce surprisingly good results. Case-control studies can be interestingly useful when the outcome studied is rare or delayed and when the exposure is difficult to randomize. However, conducting these studies can be challenging because of the great possibility of bias.^2^ These studies cannot estimate the incidence or prevalence of a disease, although they provide descriptive information about the characteristics of cases and, most importantly, an estimate of the magnitude of the association between each predicting variable and the presence or absence of disease. These estimates are expressed in the form of odds ratios, which can be approximated to the relative risk if the prevalence of the disease is not too high.^3^ ^,^ ^4^

One of the main advantages of case-control studies is the large amount of information that can be rapidly provided from a small number of subjects, which favors studies on rare outcomes and enables generation of hypotheses. Case-control studies also have limitations, such as lacking the ability to directly estimate the incidence or prevalence of the disease and the attributed risk or excess of risk. Another problem is the possibility of studying just one of many possible outcomes. However, their greatest limitation is their enormous susceptibility to bias, which results mainly from isolated sampling of cases and controls and standardization of retrospective predictive variables.^4^

The ideal case sample is the entire population (the real complete sample) or the one composed of randomly selected subjects from among those who have developed the disease under investigation. In general, sample bias becomes a real problem when the sample misrepresentation is related to the risk factor studied. In practice, case selection is usually a simple process because of the limited number of accessible subjects. However, the more challenging decisions in a case-control study relate to selection of controls. The goal is to sample controls for a population at risk of a disease that is, in other respects, similar to the case population. A good case-control study anticipates the possible forms of bias, which according to Schulz and Grimes is the most difficult task in epidemiology.^3^ If the case selection is performed in a hospital or ambulatory setting, the selection of controls should be performed in the same location; cases and controls should be paired; there should be some assurance that they are comparable with one another; cases from population-based samples can be used; and two or more control groups can even be used.^3^ ^-^ ^5^ Another important point that should be observed is the bias caused by standardization errors from the retrospective strategies used for measuring predictive variables. Two specific strategies can be used to avoid these types of bias in measuring risk factors in case-control studies: use of data registered prior to the outcome and blinding.

Although little information can be found in the PubMed and SciELO databases regarding developmental methodology in this type of study, many published papers have used the case-control design.^3^ This gives rise to some concern about the quality of these studies, and therefore justifies expanding the discussion on the methodology of case-control studies.

Considering the importance of this issue, in 2007 a group of epidemiologists, methodologists, statisticians, researchers and editors became involved in development of the STROBE statement (STrengthening the Reporting of OBservational Studies in Epidemiology). Observational research comprises several study designs and many topic areas. This group aimed to establish a checklist of items that should be included in articles reporting such kind of research. The instrument thus produced can be used for evaluating the quality of reporting observational studies and will be used in the present study.^5^ It should be noted that in reviewing the literature, we did not find any articles that evaluated published papers within the field of psychiatry using this instrument. 

## OBJECTIVE

The objective of this study was to analyze the appropriateness of the case-control designs of articles that were published in 2009, in journals that had been rated as B1 and B2 in the* Qualis* system of the Brazilian Federal Agency for the Improvement of Higher Education (Coordenação de Aperfeiçoamento de Pessoal de Nível Superior, CAPES), using the STROBE statement. Furthermore, this investigation also aimed to analyze secondary data from the distribution of the different publication types among the periodicals.

## METHODS

This was a descriptive study on articles within the field of psychiatry that were published in 2009, in Brazilian periodicals with a major ISI (Thomson Reuters Web of Knowledge, Institute for Scientific Information) international impact. The year of 2009 was selected because the current impact factors refer to articles published that year. A search was performed in the *Qualis*/CAPES database to delineate the periodicals that had psychiatric topics within their scope.

*Qualis* is a journal classification system in which the impact factors are based on the CAPES system, which is used to evaluate the scientific production of postgraduate programs.^6^ Medical journals are subclassified into three groups: Medicine I, Medicine II (which includes psychiatry) and Medicine III. This database ranks all scientific journals in eight strata (A1, A2, B1, B2, B3, B4, B5 and C), depending on their international indexing. From A1 to B2, stratification is made according to the ISI impact factor. Other Medline journals are classified as B3, Scielo journals as B4, and Lilacs journals as B5. Journals otherwise indexed are classified as C. Psychiatry journals ranked as B1 must have ISI impact factors of between 1.1 and 2.36. Journals classified as B2 must have impact factors of between 0.11 and 1.09. None of the Brazilian psychiatry journals have achieved classifications greater than B1.

In order to diminish publication bias, journals that charge for publication (i.e. *Clinics* [B1] and *Brazilian Journal of Medical and Biological Research *[B2]) did not enter the analysis. *Revista de Psiquiatria Clínica*, a B2 psychiatric journal, did not enter the evaluation because its first impact factor came in 2011. Thus, the highest qualifications found were for one journal classified as *Qualis *B1 and two journals classified as *Qualis B2* in the CAPES database. A third *Qualis *B2 journal, *Cadernos de Saúde Pública*, was excluded because among its few psychiatric articles, no abstract compatible with a case-control design was identified prior to the article analysis.

Three journals stood out partially because of their focus on the subject addressed here. Journal number 1, *Revista Brasileira de Psiquiatria*, publishes original studies from all areas of psychiatry. Its impact factor in 2009 was 1.391. Journal number 2, *Arquivos de Neuro-psiquiatria*, publishes original scientific-technological articles in the field of neurology and applied neurosciences. Its impact factor in 2009 was 0.549. Journal number 3, *Revista de Saúde Pública*, specializes in several interdisciplinary areas of public health, with emphasis on epidemiology. Its impact factor in 2009 was 1.006.

Three independent evaluators reviewed the volumes of these journals by reading the abstracts of all the articles, and then classifying the field, the design, and the type of publication of each study. Only original case-control studies within the field of psychiatry that were published in full in Brazilian periodicals with CAPES *Qualis *B1/B2 ratings in 2009 were included. Articles published in supplements of these journals were excluded, since these are only rarely peer-reviewed.

The extent to which each study fitted in with the methodology of the case-control design was evaluated in accordance with the recommendations of the STROBE statement^7^ and the theoretical basis for case-control methodology that has been described in the literature.^4^ The STROBE statement checklist (available at http://www.strobe-statement.org) was used to rate the degree of conformity. For instance, checklist items 6a and 6b refer to selection of cases and controls. Checklist item 9 refers to controlling for bias, in which authors should list all potential bias and clearly state how each of these forms of bias was dealt with. Checklist items 12a, 12b, 12c, 12d and 12e refer to statistical analysis; and checklist items 16a, 16b and 16c refer to presentation of the results. Articles were classified as "conforming" with an item if they completely complied with all subitems of the checklist, and as "non-conforming" if they did not comply with any of the subitems. All other descriptions were classified as "partially conforming".

After this selection, three evaluators independently applied the STROBE statement to all the articles, such that each article was evaluated three times by different evaluators. If there were any discrepancies in the checklist results, the evaluators formed a committee in order to reach a verdict.

*Instrument*

The STROBE statement^7^ is an instrument that was developed to ensure appropriate reporting of observational studies. It consists of 22 points that are considered by specialists to be essential for this purpose. These items involve various aspects of the study, such as the title and the abstract, as well as the detailing of facts presented in the discussion. It also includes other relevant topics, such as the financing of the project. The three types of observational design have 18 items in common, while four items are exclusively associated with each study design type: case-control, cohort and cross-sectional.

The present study took into consideration the four items that are exclusive to case-control studies. Item number one refers to the eligibility criteria of the cases and controls, the tallying sources and methods of the cases, and the selection of controls, including an explanation for how each group was chosen. Item number two refers to the efforts to address possible bias, including sampling, pairing and blinding in cases of retrospective studies. Item number three examines the suitability of the statistical methods and recommends that odds ratios should be used. These methods, particularly those that are used to control for confounding variables and manage missing data, should be clearly described. Item four refers to the presentation of results and directs attention to adjusted and non-adjusted estimates.

*Statistical analysis*

Correspondence analysis,^8^ which enables better graphic visualization of the relationships between these variables, was used to study the categorical nominal variables of the initial survey of the published articles. The general purpose of using correspondence analysis is to graphically represent the data frequencies in the form of a contingency table. The output from correspondence analysis is a two-dimensional map that makes it possible to examine associations among several categorical variables, which is not easily possible by just inspecting the frequencies or a contingency table. The distances between rows and columns are expressed using the chi-square measurement.^9^ The Kolmogorov-Smirnov test was used to investigate the normality of the distributions. The differences between averages obtained from control-case tallying were analyzed by means of the t test. The data were analyzed using the Statistical Package for the Social Sciences version 17.0 for Windows (SPSS Inc., Chicago, Illinois, USA).

## RESULTS

*Distribution of published papers*

The three journals selected generated 486 published papers in 16 volumes in 2009. The publication types were classified as original articles, letters, editorials, book reviews and special articles. The study designs were classified as case-control, clinical trial (longitudinal studies with open or closed intervention, with or without controls, with or without randomization and with or without blinding), review articles (systematic reviews and meta-analyses), case reports or case series, cohorts, other cross-sectional studies, other longitudinal studies, non-psychiatric studies (in other fields within the scope of these journals) and other designs (articles without a definite outline, such as reviews, editorials, comments, errata etc.).

**Figure 1:**
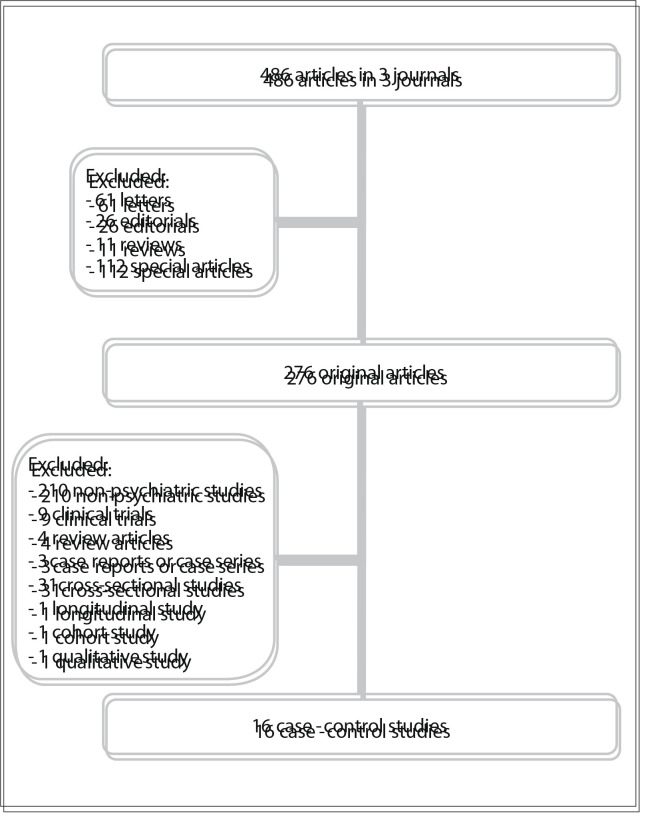
Flowchart for case-control article selection.

Sixteen published psychiatric case-control studies were found in the three selected journals (Figure 1).

*Degree of conformity of the published papers*

In relation to each item evaluated, the studies were classified as conforming (1 point), partially conforming (0.5 points) or non-conforming (0 points), as shown in Table 1. No statistically significant differences were found between the journals when the items were evaluated individually. The numbers of points for each item were summed and analyzed, and the results showed that case-control studies published in 2009 in psychiatry-specific journals obtained 2.18 points on average, compared with 1.4 points for the articles published in the neurology journal, which was a difference of 0.78 points: P= 0.031, 95% confidence interval (CI): 0.085-1.478.

**Table 1:** Conformity of case-control articles published in 2009 to case-control reporting

Journal (issue number)	Article number within issue	Conformity with items evaluated*			
		Item 1 (selection of cases and controls)	Item 2 (bias control)	Item 3 (statistical analysis)	Item 4 (presentation of results)
1 (1)	3	Partial	Partial	Partial	No
1 (1)	4	No	Partial	No	No
1 (1)	10	Partial	Partial	Partial	Partial
1 (2)	5	Yes	Yes	Partial	Yes
1 (3)	3	Yes	No	Partial	Partial
1 (3)	4	Yes	Partial	Partial	Partial
1 (3)	6	Yes	Partial	Partial	Partial
1 (4)	3	Yes	Partial	Partial	Yes
1 (4)	4	No	No	Partial	Partial
1 (4)	6	Yes	Yes	Partial	Yes
1 (4)	9	Yes	Partial	Partial	Partial
2 (1)	1	Yes	Partial	No	No
2 (2A)	1	Yes	No	No	Partial
2 (3A)	1	Yes	No	No	Partial
2 (4)	1	Yes	No	No	No
2 (4)	6	Partial	No	Partial	No

* Yes = 1, Partial = 0.5, No = 0

In relation to item 1 (selection of cases and controls), 68.8% of the articles conformed with the method, 18.8% partially conformed and 12.5% did not conform. Regarding item 2 (strategy for the control of biases), 12.5% conformed, 50% partially conformed and 37.5% did not conform. There were no published papers that completely conformed with item 3 (statistical analysis), although 68.8% partially conformed and 31.2% did not conform. In relation to item 4 (presentation of results), 18.8% conformed, 50% partially conformed and 31.2% did not conform.

*Relationship between journals and publication types*

**Figure 2:**
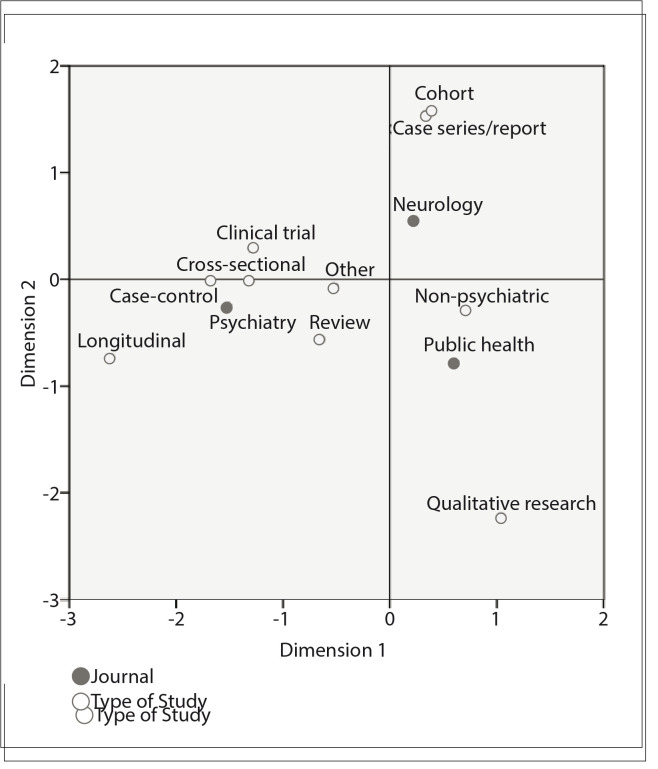
Two-dimensional derivation for correspondence analysis. Black circles represent the three journals evaluated. White circles represent the types of study (chi-square distribution of two-dimensional correspondence = 223.39; P < 0.0001).

Apart from the main analysis on the case-control studies, this investigation also tested secondary data from the distribution of the different publication types among the periodicals. As mentioned above, correspondence analysis was performed in order to explore the relationship between publication types and periodicals, and also among the different types. To view the relationship between the variables better, a correspondence analysis map was produced (Figure 2). On this map, the graphical display provides a favorable overview of the connections between the variables. It could clearly be seen that journal 2 (neurology) and journal 3 (public health) were closer together, in comparison with the distance to journal 1 (psychiatry). Moreover, clinical trials, case-control studies, review articles, other longitudinal studies and cross-sectional studies were distributed closer to journal 1 than to 2 and 3 (chi-square distribution of two-dimensional correspondence = 223.39; P < 0.0001).

## DISCUSSION

In this study, the appropriateness of the case-control methodology reporting in articles was examined. Sixteen case-control studies were retrieved from among 486 papers published in 2009 in three Brazilian journals. All of these articles, analyzed together, reached a mean score of 1,875 (on a scale from 0 to 4). The psychiatry and neurology journals differed significantly in their mean impact scores: 2.18 versus 1.4 points, respectively. This result was in accordance with the impact factor, which was greater in the psychiatry journals.

Surprisingly, none of the studies from the Brazilian journals publishing articles on psychiatry that were selected, which had higher CAPES status during this period, satisfied all of the items. None of the studies conformed completely with regard to the statistical analysis (item 3), and only 12.5% completely conformed with the item addressing the care taken in relation to potential bias (item 2). While evaluating the articles, it became clear that little attention had been given to sampling the cases and controls, controlling for possible confounding factors, matching patients and controls and choosing the most appropriate statistical method for data analysis, including the use of odds ratios, confidence intervals and sensitivity analyses. The most striking fact is that, together, these items virtually define the consistency of a case-control study.

Confusion about the term case-control study is not unique to the psychiatric literature. Only 75% of the self-declared case-control studies were found to meet the standard definitions for this design in pediatric journals,^2^ and 35% in surgery journals.^10^ In dermatology,^11^ case-control studies usually fail in relation to sample size calculations, describing and managing missing data, detailing losses from follow-up, statistical methods and the role of funding bodies in the research.

A large, well-conducted survey of the literature among major psychiatric journals (i.e. those with impact factor > 3.0) noted that the reporting of methods in case-control studies is often poor. In that survey, neuroimaging studies had the best descriptions of controlling for bias, while genetic studies had the worst.^12^

In analyzing secondary data from our sample, we also demonstrated that case-control studies, clinical trials and other cross-sectional and longitudinal studies were more frequent in psychiatry journals, while cohort studies, case reports and qualitative studies correlated more with other journals. This finding could raise new research questions and insights about what lies behind these associations. Thus, it might be asked whether case reports are uninteresting in relation to psychiatry literature; whether there is any difficulty in reporting cohort results in the Brazilian psychiatric literature; or whether the impact factor is associated with specific types of studies.

One limitation of the present study was that it was restricted to using 4 of the 22 items of the conformity evaluation instrument, i.e. the STROBE statement on case-control studies. Another important point in the analysis was that it was limited to papers published only in 2009. By increasing the sample size, it might have been possible to identify more eligible studies that were not evaluated. The non-blinding of evaluators in relation to the journals and the authors of the published papers analyzed may also have biased the results.

The fact that only Brazilian published papers were evaluated can also be considered to be a limitation, although the results from the present study are important because of its original use of the STROBE statement in psychiatric journals. Furthermore, the results can be extrapolated to countries with similar journals, and may stimulate new studies evaluating the quality of published papers on different continents.

The results show that there is a need for more research studies evaluating the appropriateness of case-control studies and other designs, with the intentions of discussing and improving the methodological quality of these studies and upgrading the clinical applicability of the results obtained from psychiatric research. Furthermore, these results should encourage clinicians who use the scientific literature in their practice to acquire basic skills for judging the validity of the articles available, and to become aware of the strengths and limitations of these articles.

## CONCLUSION

The case-control studies analyzed here did not completely conform with the four STROBE statement items for case-control design. In view of the inadequate methodology of the published studies, these findings justify focusing on research and methodology and expanding the investigations on adherence of studies to their designs.
